# Clinical Characteristics and Cardiac Magnetic Resonance Profile of *PKP2* Variant–Positive Patients With a Hypertrophic Cardiomyopathy Phenotype

**DOI:** 10.1016/j.jacadv.2026.102640

**Published:** 2026-03-05

**Authors:** Matteo Castrichini, Ramin Garmany, Konstantinos C. Siontis, Darrell B. Newman, Jeffrey B. Geske, Steve R. Ommen, Jay Schneider, Leslie T. Cooper, Michael J. Ackerman, John R. Giudicessi

**Affiliations:** a Department of Cardiovascular Medicine, Mayo Clinic, Rochester, Minnesota, USA; b Mayo Clinic Medical Scientist Training Program, Mayo Clinic Graduate School of Biomedical Sciences, Mayo Clinic Alix School of Medicine, Rochester, Minnesota, USA; c Department of Cardiovascular Medicine, Mayo Clinic, Jacksonville, Florida, USA; d Department of Molecular Pharmacology and Experimental Therapeutics, Mayo Clinic, Rochester, Minnesota, USA; e Department of Pediatrics (Division of Pediatric Cardiology), Rochester, Minnesota, USA

**Keywords:** arrhythmogenic cardiomyopathy, hypertrophic cardiomyopathy, genetics, PKP2

Pathogenic/likely pathogenic (P/LP) variants in *PKP2*-encoded plakophilin-2 (PKP2) are the most common cause of arrhythmogenic cardiomyopathy (ACM), a disease characterized by fibrofatty replacement of the myocardium, ventricular arrhythmias, and sudden cardiac arrest.^1^ Traditionally considered a right-dominant or biventricular disease, PKP2-related ACM often presents in young individuals with typical electrocardiographic and imaging features.^1^ Conversely, hypertrophic cardiomyopathy (HCM), defined by unexplained myocardial hypertrophy, is typically caused by sarcomeric gene mutations. Nevertheless, overlapping phenotypes have been reported in genes outside the sarcomeric machinery, such as *PLN* and *JPH2*,^2^^,^^3^, which are involved in calcium handling. As a desmosomal protein involved in cell-cell adhesion and intracellular signaling, PKP2 also plays a role in calcium homeostasis^4^ which raises the question of whether P/LP variants in PKP2 could disrupt calcium signaling, particularly in permissive genetic backgrounds,^5^ in a way that could promote hypertrophic remodeling.

As such, we sought to determine the prevalence of left ventricular hypertrophy (LVH) in a large cohort of PKP2 P/LP variant-positive patients and compare the clinical and imaging features, as well as arrhythmic outcomes, between PKP2 P/LP variant-positive individuals with LVH/HCM vs those with ACM. The study was approved by the institutional review board, and all participants provided informed consent.

**What is the clinical question being addressed?**

What is the prevalence of HCM in patients with a disease-causative variant in PKP2?

**What is the main finding?**

Eight percent of PKP2 variant-positive patients met the HCM criteria, but the predominance of basal septal hypertrophy and low arrhythmic burden question PKP2’s ability to function as a primary HCM gene.

We performed a retrospective cohort analysis of patients enrolled in our institutional inherited ACM registry between January 2015 and May 2025. All patients had undergone genetic testing and were confirmed to carry a P/LP variant in the *PKP2* gene. HCM was defined as a maximal left ventricular wall thickness >15 mm (or >13 mm in first-degree relatives), not due to secondary causes. ACM was diagnosed based on the 2020 Padua criteria, incorporating imaging, electrocardiogram, and arrhythmic features.^1^ Demographic, clinical, electrocardiogram, and imaging data were extracted from the electronic medical record. Cardiac magnetic resonance (CMR) findings included late gadolinium enhancement (LGE) presence and distribution. Major ventricular arrhythmic (MVA) outcomes included sustained ventricular tachycardia, ventricular fibrillation, sudden cardiac arrest, with or without appropriate implantable cardioverter defibrillator therapy. Continuous variables were presented as mean ± SD and compared using Student’s t-test when normally distributed. Normality was assessed using the Shapiro-Wilk test and visual inspection of histograms. Skewed data were presented as median and compared using the Wilcoxon rank-sum test. Categorical variables were presented as count (percentage) and compared using chi-square or Fisher exact test, as appropriate. A *P* value <0.05 was considered statistically significant.

Among the 134 PKP2 P/LP-positive patients, 74 (55%) showed an overt cardiomyopathy phenotype: 64 (48%) with ACM and unexpectedly 10 (8%) with an HCM phenotype. Interestingly, most HCM (90%) cases had a sigmoid septal morphology. All 10 patients underwent genetic testing that covered all ClinGen-adjudicated HCM genes with definitive, strong, or moderate evidence. Importantly, patients with HCM and ACM phenotypes harbored overlapping P/LP *PKP2* variants, with no segregation by variant type or gene region between phenotypes. HCM patients were significantly older (64 ± 7 vs 45 ± 17 years, *P* = 0.001) and more frequently had a diagnosis of hypertension (80% vs 18%, *P* = 0.003). Moreover, HCM patients had higher systolic and diastolic blood pressure, more sleep apnea and diabetes, and less chronic kidney disease than ACM patients. Electrocardiogram-detected LVH was only observed in HCM patients (50% vs 0%, *P* < 0.001). A family history of cardiomyopathy was more frequent in ACM (44% vs 10%, *P* = 0.037). Syncope, atrial fibrillation, and atrioventricular block rates were similar between groups. HCM patients were less likely to have nonsustained ventricular tachycardia (17% vs 70%, *P* = 0.017) and anterior T wave inversion (10% vs 60%, *P* = 0.005). The prevalence of MVA were observed in only 1 HCM patient (10%) compared to 38 ACM patients (59%, *P* = 0.004). Implantable cardioverter defibrillator implantation was more frequent in ACM (79% vs 40%, *P* = 0.015). As expected, the HCM group had significantly greater maximal left ventricular wall thickness (17 ± 2 mm vs 9 ± 2 mm, *P* < 0.001) and higher left ventricular outflow tract gradients (57 ± 48 mm Hg vs 0 mm Hg, *P* < 0.001). Moderate or greater mitral regurgitation was more common in HCM (40% vs 5%, *P* = 0.008). There was no significant difference in left ventricular ejection fraction or left ventricular end-diastolic volume. (Table 1). Importantly, none of the patients with an HCM phenotype was related to any of the individuals with an ACM phenotype, with no overlap in pedigrees between groups. Seven of 10 patients (70%) had left ventricular outflow tract obstruction, and 3 myectomy specimens showed hypertrophy, mild disarray, and fibrosis.

**Table 1:** Baseline Characteristic of the PKP2 Variant–Positive Phenotype Positive Patients, Comparing the Patients With HCM Phenotype and ACM Phenotype

	Total (N = 74)	HCM Phenotype (n = 10)	ACM Phenotype (n = 64)	*P* Value
Male, n (%)	35 (47.3)	5 (50.0)	34 (53.1)	0.85
Age, y	**48 ± 17**	**64 ± 7**	**45 ± 17**	**0.001**
White, n (%)	67 (90.5)	8 (80.0)	59 (92.2)	0.14
Family history of sudden cardiac arrest, n (%)	23 (31.1)	3 (30.0)	20 (31.3)	0.61
Family history of cardiomyopathy, n (%)	**29 (39.2)**	**1 (10.0)**	**28 (43.8)**	**0.037**
History of syncope, n (%)	25 (33.8)	2 (20.0)	23 (35.9)	0.25
History of hypertension, n (%)	**26 (35.1)**	**8 (80.0)**	**18 (28.1)**	**0.003**
Systolic blood pressure (mm Hg)	**118 ± 18**	**137 ± 23**	**116 ± 16**	**<0.001**
Diastolic blood pressure (mm Hg)	**74 ± 11**	**73 ± 11**	**83 ± 13**	**0.005**
Obstructive sleep apnea, n (%)	**22 (29.7)**	**4 (40.0)**	**18 (28.1)**	**0.06**
Diabetes, n (%)	9 (12.2)	2 (20.0)	7 (10.9)	0.14
BMI (kg/m^2^)	27 ± 8	27 ± 8	28 ± 6	0.82
Chronic kidney disease, n (%)	24 (32.4)	1 (10.0)	23 (35.9)	0.43
LVH on ECG, n (%)	**5 (6.8)**	**5 (50.0)**	**0 (0.0)**	**<0.001**
Atrioventricular block, n (%)	16 (21.6)	2 (20.0)	14 (21.9)	0.53
Atrial fibrillation, n (%)	23 (31.1)	5 (50.0)	18 (28.1)	0.21
NSVT, n (%)	**39 (52.7)**	**1 (10.0)**	**38 (59.4)**	**0.017**
Anterior T negative waves, n (%)	**32 (43.2)**	**1 (10.0)**	**31 (48.4)**	**0.005**
Inferior T negative waves, n (%)	13 (17.6)	1 (10.0)	12 (18.8)	0.31
Lateral T negative waves, n (%)	19 (25.7)	3 (30.0)	16 (25.0)	0.62
Stroke, (n %)	3 (4.1)	2 (20.0)	1 (1.6)	0.08
Hyperlipidemia, n (%)	12 (16.2)	4 (40.0)	8 (12.5)	0.18
ICD, n (%)	**55 (74.3)**	**4 (40.0)**	**51 (79.7)**	**0.015**
Major VA, n (%)	**39 (52.7)**	**1 (10.0)**	**38 (59.4)**	**0.004**
Advanced HF therapies, n (%)	11 (14.9)	0 (0.0)	11 (17.2)	0.18
LVEF, %	56 ± 11	57 ± 11	56 ± 11	0.79
LVEDV, mL	140 ± 41	126 ± 33	143 ± 42	0.21
RVEDV, mL	**212 ± 73**	**126 ± 33**	**226 ± 67**	**<0.001**
MLVWT, mm	**10 ± 3**	**17 ± 2**	**9 ± 2**	**<0.001**
Moderate or greater MR, n (%)	**7 (9.5)**	**4 (40.0)**	**3 (4.7)**	**0.008**
LVOT gradient, mm Hg	**8 ± 26**	**57 ± 48**	**0**	**<0.001**
Presence of LGE, n (%)	26/53 (49.1)	5/8 (62.5)	21/45 (46.7)	0.41
Inferolateral LGE, n (%)	9/53 (17.0)	1/8 (12.5)	8/45 (17.8)	0.65
Septal LGE, n (%)	**10/53 (18.9)**	**5/8 (62.5)**	**5/45 (11.1)**	**0.004**
RV LGE, n (%)	**16/53 (30.2)**	**0/8 (0.0)**	**16/45 (35.6)**	**0.037**

Values are mean ± SD or n (%). Percentages are reported with 1 digit to the right of the decimal. *P* values >0.10 are reported with 2 digits to the right of the decimal. Statistically significant comparisons appear in bold text.

ACM = arrhythmogenic cardiomyopathy; BMI = body mass index; ECG = electrocardiogram; HCM = hypertrophic cardiomyopathy; HF = heart failure; ICD = implantable cardioverter defibrillator; LGE = late gadolinium enhancement; LVEDV = left ventricular end-diastolic volume; LVEF = left ventricular ejection fraction; LVH = left ventricular hypertrophy; LVOT = left ventricular outflow tract; MLVWT = maximum left ventricular wall thickness; MR = mitral regurgitation; NSVT = non-sustained ventricular tachycardia; RV = right ventricle; RVEDV = right ventricular end-diastolic volume; VA = ventricular arrhythmia.

On CMR, septal LGE was markedly more frequent in HCM (63% vs 11%, *P* = 0.004), whereas right ventricular LGE was seen exclusively in ACM (36% vs 0%, *P* = 0.037). Inferolateral LGE was observed in both groups without significant difference (Table 1). Overall, 80% of the HCM patients had mild LGE burden on CMR (<10% left ventricular mass).

This study identifies a meaningful subset (8%) of PKP2 P/LP variant–positive patients who meet the diagnostic criteria for HCM (13.5% among patients with overt phenotype) with no clinical features of ACM. Compared to ACM patients, those with HCM phenotype were older, had a higher prevalence of hypertension, and exhibited septal hypertrophy and LGE, accompanied by significantly lower arrhythmic burden. In contrast, patients with a classical ACM phenotype exhibited a significantly higher burden of ventricular arrhythmias, anterior T-wave inversions, and right ventricular LGE. Interestingly, no PKP2 P/LP variant-positive individual was found to have evidence of concomitant ACM and HCM.

Given the absence of cosegregation of an HCM phenotype with P/LP PKP2 variants, it seems unlikely that PKP2 functions as a self-sufficient LVH/HCM-susceptibility gene. However, the higher-than-expected prevalence of a sigmoid HCM phenotype (8%) in PKP2 variant–positive patients together with the degree of outflow obstruction requiring septal myectomy, raise the possibility that perturbed calcium handling from PKP2 loss of function may act as a contributing factor in individuals with a permissive genetic background (eg, elevated HCM polygenic risk score) and environmental risks such as hypertension or obstructive sleep apnea. Notably, although the ACM group had a high rate of MVA events, the HCM group had only 1 (10%), suggesting that PKP2 variant–positive patients who develop an HCM phenotype may be relatively protected from an ACM standpoint. Additional studies are needed to clarify whether HCM phenotype or polygenic susceptibility lowers ACM penetrance/risk. Finally, given the exploratory and hypothesis-generating nature of these analyses, no formal adjustment for multiple comparisons was performed, and findings should be interpreted with appropriate caution.

In conclusion, in our single-center cohort, 8% of all individuals with P/LP PKP2 variants and 13.5% of those with overt cardiac disease demonstrated an HCM phenotype without right ventricular involvement. Mechanistically, this raises questions about whether PKP2 haploinsufficiency directly influences hypertrophic signaling through calcium-handling abnormalities or whether the overlap reflects ascertainment bias, given the population frequencies of HCM (1:500) and PKP2 loss-of-function variants (1:695). Prospective studies are required to validate these observations.

## Funding support and author disclosures

This work was supported by the Mayo Clinic Windland Smith Rice Comprehensive Sudden Cardiac Death Program (Dr Ackerman) and the Paul and Ruby Tsai and Family Fund for Hypertrophic Cardiomyopathy Research (Drs Ackerman and Giudicessi). Dr Ackerman is a consultant for 10.13039/100000046Abbott, ARMGO Pharma, 10.13039/100008497Boston Scientific, Bristol Myers Squibb, 10.13039/100010905Illumina, 10.13039/100020388Invitae, 10.13039/100004374Medtronic, Tenaya Therapeutics, and UpToDate; he and Mayo Clinic are involved in an equity/royalty relationship with AliveCor, Anumana, Prolaio, Solid Biosciences, and Thryv Therapeutics. Dr Giudicessi is a consultant for Avidity Biosciences, Citizen Health and Nuevocor Therapeutics; and he is the principal investigator for clinical trials sponsored by Tenaya Therapeutics and Solid Biosciences. All other authors have reported that they have no relationships relevant to the contents of this paper to disclose.
